# Recent Advances in Neuronal-like Differentiation of Mesenchymal Stem/Stromal Cells

**DOI:** 10.3390/ijms27219902

**Published:** 2026-11-05

**Authors:** Ivana Roberta Romano, Floriana D’angeli, Grazia Maugeri, Giuseppe Musumeci, Serena Tamburino, Giuseppe Angelo Giovanni Lombardo, Rosario Giuffrida, Giuliana Mannino, Debora Lo Furno

**Affiliations:** 1 Department of Biomedical and Biotechnological Sciences, University of Catania, 95123 Catania, Italy; ivanarobertaromano@yahoo.it (I.R.R.); grazia.maugeri1@unict.it (G.M.); giuseppe.musumeci@unict.it (G.M.); giuffros@unict.it (R.G.); lofurno@unict.it (D.L.F.); 2 Department of Medicine and Surgery, University of Enna “Kore”, 94100 Enna, Italy; floriana.dangeli@unikore.it (F.D.); giuseppe.lombardo@unikore.it (G.A.G.L.); 3 Chi.Pla Chirurgia Plastica, Via Suor Maria Mazzarello, 54, 95128 Catania, Italy

**Keywords:** mesenchymal stem/stromal cells, neuronal differentiation, Neuroregeneration, regenerative medicine, stem cell therapy, neurodegenerative diseases

## Abstract

Mesenchymal stem/stromal cells (MSCs) have been widely investigated in recent decades because of their multipotent differentiation abilities. In fact, MSCs are able to differentiate not only into mesodermal cell lines (adipocytes, chondrocytes, osteocytes), but also into neuronal and glial cells. MSCs can be derived from different sources, such as bone marrow, adipose tissue, peripheral blood and various neonatal birth-associated tissues. They are suitable candidates in cell-based approaches for both autologous and heterologous transplantation in the field of regenerative medicine. Unlike other types of stem cells, MSCs raise no ethical issues and feature low risks of tumorigenesis after transplantation into humans or animals. In addition, they may exert beneficial effects due to their paracrine actions which help tissue recovery. Numerous studies describe effects obtained following MSC treatments in a variety of human pathologies, including nervous system diseases. The nervous tissue is characterized by very limited recovery properties, likely because of a scarce presence of stem cell niches, which are confined to the dentate gyrus of the hippocampus and the subventricular zone of the lateral ventricles. These niches cannot be able to counteract the neuronal loss after damage or degenerative events. Many efforts have been made to obtain functional neuronal differentiated MSCs to be used for transplantation. However, evidence of complete neuronal maturation and functional integration of neuronal-like MSCs remains limited, and clinical studies have used undifferentiated MSCs rather than neuronal-like cell populations. In this review, we mainly focus on various differentiation protocols that have been adopted for in vitro studies, taking also into account the underlying pathways involved.

## 1. Introduction

**Figure 1:**
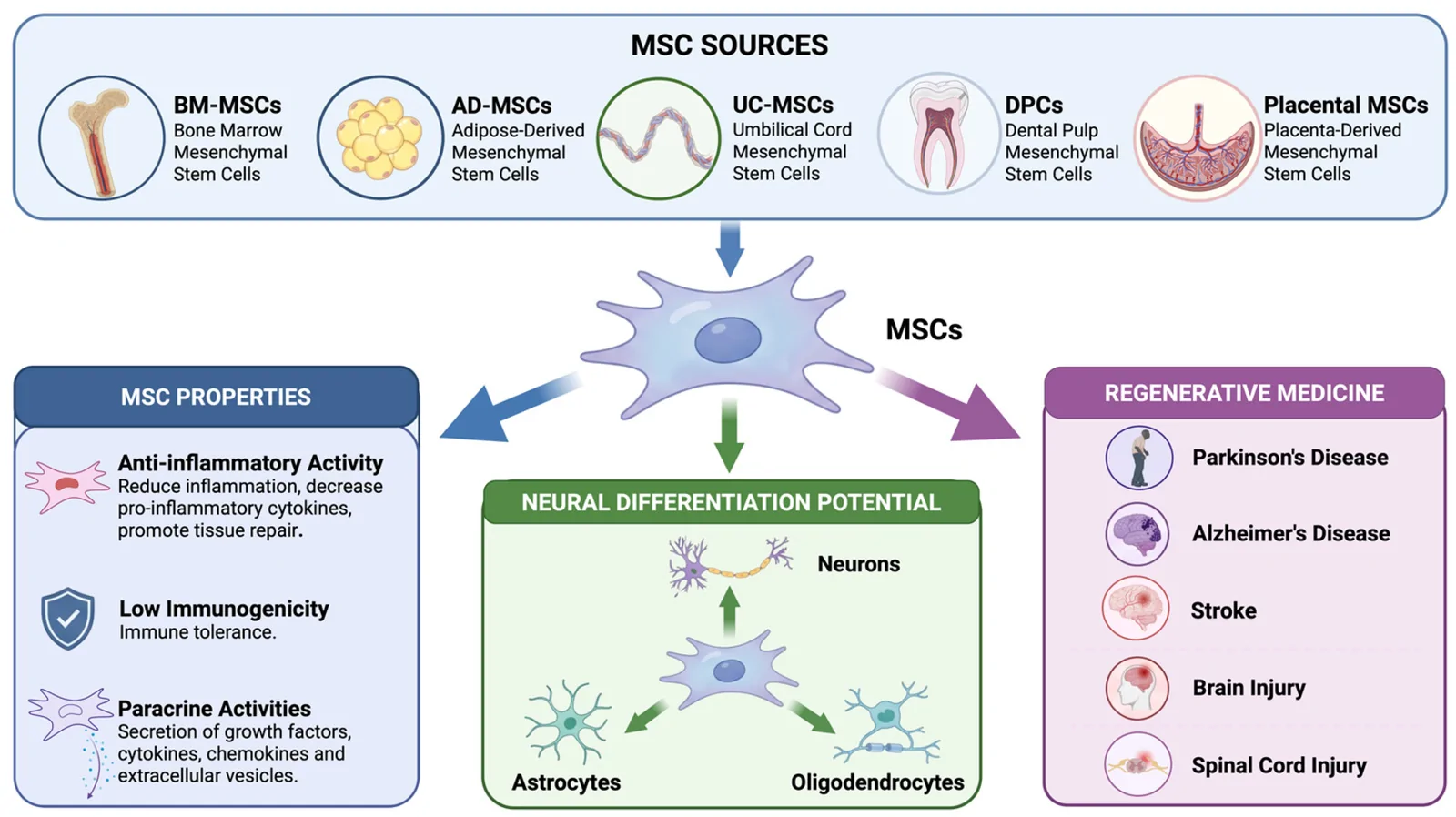
Mesenchymal Stem/Stromal Cells for Neuroregeneration: Sources, Biological Properties and Neural Differentiation Potential.

Mesenchymal stem/stromal cells (MSCs) are multipotent adult stem cells that have attracted considerable attention in regenerative medicine because of their self-renewal capacity, multilineage differentiation potential, immunomodulatory properties, and favorable safety profile. Initially identified in bone marrow by Friedenstein and colleagues and later characterized as a distinct stromal stem cell population, MSCs can be isolated from a variety of adult and perinatal tissues, including bone marrow, adipose tissue, umbilical cord, Wharton’s jelly, placenta, amniotic membrane, dental pulp, and peripheral blood. Their ease of isolation and expansion, together with the absence of major ethical concerns and the low risk of tumorigenesis compared with embryonic stem cells and induced pluripotent stem cells, have made MSCs one of the most extensively investigated cell types for clinical applications in tissue engineering and regenerative medicine [1,2,3,4,5,6]. Beyond their well-established ability to differentiate into mesodermal lineages such as osteoblasts, adipocytes, and chondrocytes, accumulating evidence indicates that MSCs possess remarkable phenotypic plasticity and can acquire characteristics of ectodermal and endodermal cell types under appropriate experimental conditions. In particular, numerous studies have demonstrated the ability of MSCs to undergo neuronal-like differentiation in vitro, expressing neural progenitor, neuronal, and glial markers and, in some cases, exhibiting electrophysiological properties resembling those of mature neurons [7,8,9,10,11,12]. This neurogenic potential, together with their potent paracrine activity and immunoregulatory functions, has generated increasing interest in the development of MSC-based approaches for neurological disorders (Figure 1).

Neurological diseases constitute one of the leading causes of disability and mortality worldwide. Conditions such as ischemic stroke, traumatic brain injury, spinal cord injury, Parkinson’s disease (PD), Alzheimer’s disease (AD), Huntington’s disease, and amyotrophic lateral sclerosis (ALS) are characterized by progressive neuronal loss and limited regenerative capacity [13,14]. Unlike many other tissues, the adult central nervous system exhibits only restricted endogenous repair mechanisms. Neurogenesis persists throughout life but is largely confined to specialized niches, namely the subventricular zone of the lateral ventricles and the subgranular zone of the dentate gyrus [15,16,17]. Although neural stem cells residing within these regions contribute to physiological neuronal turnover, their regenerative potential is insufficient to compensate for the extensive neuronal degeneration associated with traumatic, ischemic, or neurodegenerative conditions [5].

The possibility of generating neuronal cells from MSCs therefore represents an attractive strategy for both cell replacement therapies and neuroprotective interventions [18,19]. Over the last two decades, a wide range of differentiation protocols have been developed to improve the efficiency and stability of MSC neurogenic conversion. Early protocols relied primarily on chemical induction agents, including β-mercaptoethanol, dimethyl sulfoxide (DMSO), and butylated hydroxyanisole (BHA), whereas more recent approaches employ combinations of neurotrophic factors, developmental morphogens, small molecules, genetic engineering techniques, extracellular matrix components, biomaterials, and three-dimensional (3D) culture systems designed to recapitulate key aspects of neural development [20,21,22,23].

Recent advances in stem cell biology, single-cell transcriptomics, epigenetic regulation, and tissue engineering have substantially improved our understanding of the molecular mechanisms underlying MSC neuronal differentiation [24,25].

In this review, we summarize the current knowledge regarding neuronal-like differentiation of MSCs, with particular emphasis on advances reported during the last ten years. We discuss the biological basis of MSC neuroplasticity, show the principal differentiation strategies currently available, and evaluate their potential applications in regenerative neurology. Finally, we address the major limitations and future perspectives that must be overcome to facilitate the clinical translation of MSC-derived neuronal products.

###  Literature Search Strategy and Study Selection

This review aimed to provide an overview of advances in MSC neuron-like differentiation, with particular emphasis on research published over the last decade (from 2016 to 2026) and its implications for regenerative medicine and neurological disorders.

The literature search was conducted primarily through PubMed/MEDLINE and complemented by searches using Google Scholar and direct consultation of scientific publishers’ websites, including Nature. These complementary sources were used to identify additional relevant publications and explore recent developments in the field.

The search strategy was based on combinations of keywords reflecting the main topics of the review, including “mesenchymal stem cells”, “MSC” AND “cell therapy” AND “neurodegenerative disease”, “neural differentiation”, “retina” AND “MSC”, “retinal ganglion cells” OR “photoreceptor” AND “MSC”, and “MSC secretome”. Additional terms relating to neuronal differentiation, molecular signaling pathways, specific neuronal phenotypes, extracellular vesicles, biomaterials, and regenerative applications were considered according to the topics investigated. Because this is a narrative review, study selection was purposive rather than exhaustive.

The selection of publications was guided by their relevance, their contribution to understanding the molecular and cellular mechanisms underlying MSC neuronal differentiation, and their significance for the development of regenerative strategies. Original research articles, relevant reviews, preclinical investigations, and clinical studies were considered to provide complementary perspectives on the biological mechanisms, experimental approaches, therapeutic potential, and translational challenges associated with MSC-based therapies.

The evidence was synthesized qualitatively, without quantitative pooling or meta-analysis, in keeping with the narrative nature of this review.

## 2. Molecular Basis of MSC Neurodifferentiation

The neuronal differentiation of MSCs is a highly complex and tightly regulated process involving the coordinated interaction of extracellular signals, intracellular signaling cascades, transcription factors, epigenetic regulators, and microenvironmental cues. Although MSCs are traditionally considered mesoderm-derived progenitors, accumulating evidence suggests that they retain a remarkable degree of developmental plasticity, enabling their conversion toward neural phenotypes under appropriate experimental conditions [8,26,27,28]. Recent advances in transcriptomic and proteomic analyses have revealed that neurodifferentiation is not driven by a single pathway but rather by the dynamic integration of multiple signaling networks that collectively regulate lineage commitment, neuronal maturation, and functional specialization [29,30].

MSC neurogenic protocols largely attempt to recapitulate embryonic neural development through canonical signaling pathways. Among the pathways, Wnt/β-catenin signaling regulates neural patterning and progenitor expansion [31]. Thus, in MSC neurodifferentiation, Wnt signaling plays a central role in regulating neural lineage specification, neuronal maturation, and axonal development [32]. During embryonic neurogenesis, Wnt proteins control the balance between stem cell maintenance and neuronal differentiation, and similar mechanisms appear to operate during MSC neural differentiation [33,34]. Activation of canonical Wnt/β-catenin signaling promotes the expression of neurogenic genes, enhances neuronal marker expression, and supports neurite outgrowth [35]. Recent evidence demonstrated that Wnt5a overexpression significantly increases the neuronal differentiation efficiency of bone marrow-derived MSCs (BM-MSCs) through activation of the phosphatidylinositol-3-kinase/protein kinase B (PI3K/Akt) pathway and subsequent upregulation of neuronal markers including microtubule-associated protein 2 (MAP2) and βIII-tubulin (TUBB3) [36].

Notch signaling is another highly conserved pathway involved in the regulation of stem cell maintenance and neural differentiation. In neural development, Notch activity preserves progenitor cell populations while suppressing premature neuronal differentiation [37,38]. During MSC neurodifferentiation, modulation of Notch signaling appears to be essential for the transition from a proliferative mesenchymal phenotype toward a neural fate [39]. Downregulation of Notch signaling facilitates the expression of proneural transcription factors such as achaete-scute family bHLH transcription factor 1 (ASCL1 or Mash1), Neurogenin-1 (Ngn1), and Neurogenin-2 (Ngn2), whereas persistent Notch activation tends to maintain stemness characteristics [40,41]. Recent studies using genetically modified MSCs have demonstrated that controlled inhibition of Notch signaling significantly enhances neuronal marker expression and promotes acquisition of neuron-like morphology [42].

The Sonic Hedgehog (SHH) pathway plays a crucial role in neural tube patterning, ventral midbrain specification and dopaminergic neuron identity during embryogenesis [43]. In MSC differentiation protocols, SHH is frequently combined with fibroblast growth factor 8 (FGF8), brain-derived neurotrophic factor (BDNF), and glial cell-derived neurotrophic factor (GDNF) to promote dopaminergic neuronal differentiation. Activation of SHH signaling induces the expression of key transcription factors such as LIM homeobox transcription factor 1 alpha (LMX1A), nuclear receptor related 1 protein (NURR1), and Pentraxin-related protein (PITX3), which are required for dopaminergic lineage commitment. Consequently, SHH-based protocols have become among the most commonly used strategies for generating dopaminergic neuron-like cells from MSCs for potential applications in Parkinson’s disease [44,45,46].

The PI3K/Akt pathway is a major regulator of cell survival, proliferation, differentiation, and metabolism. Increasing evidence indicates that PI3K/Akt signaling contributes significantly to MSC neurodifferentiation by promoting neuronal survival and facilitating the expression of neural-specific genes [47]. Activation of PI3K/Akt has been observed following stimulation with neurotrophic factors such as BDNF and nerve growth factor (NGF), as well as following Wnt5a-mediated neuronal induction [36]. Pharmacological inhibition of PI3K signaling results in reduced neuronal differentiation efficiency and decreased expression of Neuronal nuclei (NeuN), Neuron-specific enolase (NSE), MAP2, and tyrosine hydroxylase (TH), highlighting the importance of this pathway in neurogenic commitment [48].

The mitogen-activated protein kinase/extracellular signal-regulated kinase (MAPK/ERK) pathway is another critical mediator of MSC neurodifferentiation. Activation of ERK signaling by growth factors including epidermal growth factor (EGF), basic fibroblast growth factor (bFGF), BDNF and NGF promotes neuronal commitment and supports the differentiation process through regulation of expression of neuronal genes [49]. Some studies have demonstrated that modulation of MAPK/ERK activity influences neuronal subtype specification and contributes to the acquisition of characteristics of mature neurons [50,51].

Some evidence demonstrated that transcription factors such as ASCL1, Neuronal Differentiation 1 (NeuroD1), Ngn1, Ngn2, SRY-box 2 (Sox2), paired box 6 (Pax6), NURR1, and LMX1A act as pioneer regulators capable of remodeling chromatin accessibility and initiating neuronal gene expression programs in several cell types such as induced pluripotent stem cells, neural progenitors, fibroblasts, and MSCs [32,52,53,54,55,56].

Collectively, these findings indicate that MSC neurodifferentiation results from the coordinated activity of multiple signaling pathways and regulatory networks.

**Figure 2:**
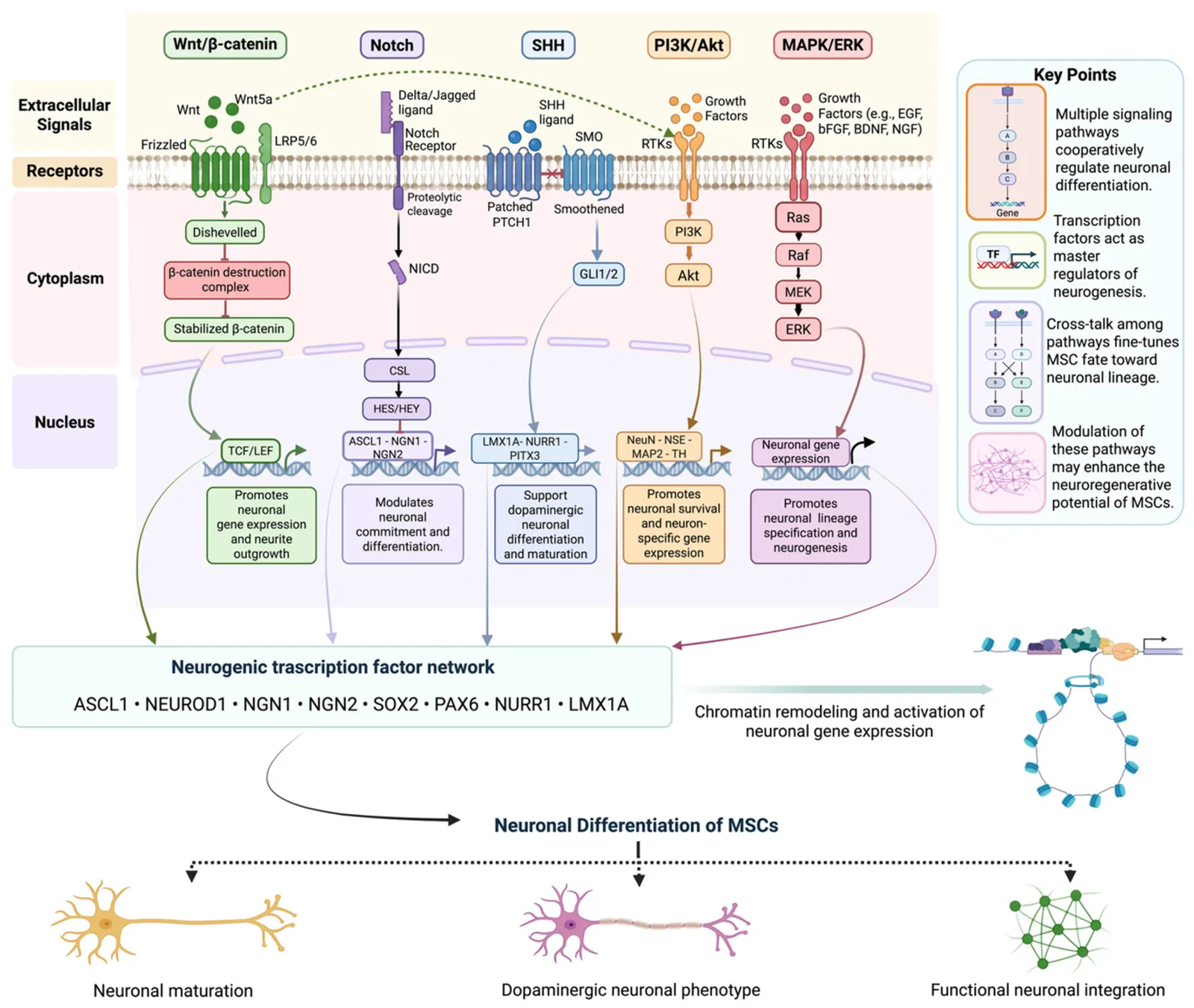
Hypothetical model of signaling pathways potentially associated with neuronal-like differentiation of mesenchymal stem/stromal cells (MSCs). Neuronal differentiation of MSCs is likely regulated by the coordinated activity of Wnt/β-catenin, Notch, Sonic Hedgehog (SHH), PI3K/AKT, and MAPK/ERK signaling pathways. Canonical Wnt signaling inhibits the β-catenin destruction complex, allowing stabilized β-catenin to activate TCF/LEF-dependent transcription factors, promoting neuronal gene expression and neurite outgrowth. Wnt5a might also support neuronal differentiation through PI3K/AKT pathway activation. Notch signaling modulates neuronal commitment: NICD associates with RBPJ/CSL and induces HES/HEY factors, which repress proneural genes; consequently, Notch downregulation would facilitate neuronal differentiation. Binding of SHH to PTCH1 relieves PTCH1-mediated inhibition of SMO, enabling GLI1/2 activation and the expression of dopaminergic determinants, including LMX1A, NURR1, and PITX3. Growth factors and neurotrophins activate PI3K/AKT and MAPK/ERK signaling, supporting neuronal survival, neural-specific gene expression and neurogenesis. NeuN, NSE, MAP2, and TH are shown as downstream markers of neuronal differentiation associated with PI3K/AKT activity. These pathways would converge on a neurogenic transcription-factor network, including ASCL1, NEUROD1, NGN1, NGN2, SOX2, PAX6, NURR1, and LMX1A, that contributes to chromatin remodeling and activation of neuronal gene-expression programs, ultimately promoting neuronal maturation, dopaminergic phenotype specification, and functional specialization. Arrows indicate activation, blunt-ended lines indicate inhibition, and the crossed inhibitory connection between PTCH1 and SMO indicates relief of inhibition following SHH binding.

Thus, the balance between pro-neurogenic signaling, maintenance of stemness, cell survival, and subtype specification is determined by the dynamic interaction among these pathways rather than by single molecular mechanisms. This integrated signaling architecture ultimately provides the molecular basis for the transition of MSCs from a mesenchymal state toward a neuron-like phenotype and highlights the importance of pathway crosstalk in determining the efficiency, maturation, and functional characteristics of MSC-derived neural cells (Figure 2). However, the effects of pathway blockade at different induction stages remain poorly investigated.

Furthermore, responses could also depend on MSC source and experimental conditions: comparative studies report differences in neural-marker expression and induction kinetics among MSCs from different tissues [8], while dose dependence has been demonstrated for Wnt7a [34]. Moreover, extracellular matrix properties, including stiffness, topography, adhesive cues, and 3D culture, can influence neural-like outcomes in MSCs [52,54,57,58]. However, their direct interactions with the reported pathways remain to be established. At the gene-regulatory level, MSC studies associate Wnt/TCF activity with Tlx3 expression [32], report NRSF downregulation during chemical neural induction [50] and investigate neuronal differentiation following targeted hypermethylation of Salvador/Warts/Hippo pathway genes [51]. These findings do not establish a common causal transcriptional or epigenetic program across MSC models.

## 3. Neuronal-Differentiation Protocols of MSCs

Over the past decade, the neurogenic differentiation of MSCs has progressively been redefined as an emergent process governed by the integration of biochemical cues, biophysical forces, and cellular engineering strategies, rather than a response driven solely by soluble induction cocktails. A pivotal conceptual advance was provided by Engler et al. (2006), who demonstrated that extracellular matrix stiffness is a key determinant of stem cell lineage specification [57]. In particular, substrates mimicking the elasticity of brain tissue were shown to preferentially promote neurogenic programs in MSCs, thereby establishing matrix mechanics as an instructive regulator of cell fate and introducing the concept that cells actively interpret and “remember” the mechanical properties of their microenvironment.

This framework was subsequently refined by the identification of core mechanotransduction pathways. Dupont et al. (2011) demonstrated that the transcriptional co-activators YAP and TAZ, downstream effectors of the Hippo pathway, act as central sensors of cytoskeletal tension. In response to mechanical cues, YAP/TAZ shuttle between the cytoplasm and nucleus, where they regulate gene expression programs associated with proliferation, lineage commitment, and differentiation. On soft, brain-like substrates, YAP/TAZ are predominantly excluded from the nucleus, thereby favoring differentiation-associated transcriptional states, including neurogenic programs. Importantly, this mechanosensitive axis interfaces with canonical developmental signaling pathways such as Wnt/β-catenin, Notch, and Sonic Hedgehog, which collectively shape chromatin accessibility and neuronal gene expression [58].

More recently, the concept of biophysical regulation has expanded beyond matrix stiffness to include the nanoscale organization of the plasma membrane and focal adhesion dynamics. In this context, Jia et al. (2022) demonstrated that adaptive liquid interfaces composed of protein nanofibrils can induce spontaneous neuronal-like differentiation of human BM-MSCs through lipid raft clustering and focal adhesion kinase (FAK)-dependent signaling. This process leads to the upregulation of neuronal markers such as βIII-tubulin and MAP2 even in the absence of classical neurogenic induction media. Notably, pharmacological disruption of lipid raft integrity or FAK signaling almost completely abolishes neuronal commitment, highlighting a direct causal role of membrane nanodomain organization in lineage specification. These findings support a view of the plasma membrane as a dynamic signaling platform that integrates mechanical and biochemical information to regulate cell fate decisions [59].

Consistent with this concept, Panda et al. (2021) further demonstrated that substrate physicochemical properties can direct lineage specification toward electrophysiologically distinct neural phenotypes. Using engineered electroactive substrates with tunable surface functionalities, the authors showed that human BM-MSCs could be selectively guided toward either neuron-like or glial-like cells depending on the material chemistry. Importantly, differentiated cells exhibited distinct electrophysiological characteristics, including differences in membrane potential and ion channel activity, indicating that substrate engineering influences not only lineage-specific marker expression but also functional maturation. These observations reinforce the concept that rational biomaterial design can actively instruct neural differentiation by integrating mechanical, electrical, and biochemical cues [60].

Parallel advances in biomaterial engineering have increasingly focused on reproducing key features of the neural extracellular matrix to enhance neuronal maturation. In this regard, Jana et al. (2024) developed an engineered self-assembling peptide hydrogel combined with a neural differentiation medium to generate functionally mature neurons from human umbilical cord-derived MSCs (UC-MSCs). Compared with conventional induction protocols, the 3D hydrogel microenvironment significantly enhanced neuronal morphology, neurite extension, expression of mature neuronal markers, and electrophysiological functionality. Importantly, transplantation of the differentiated cells into a rat model of traumatic brain injury promoted neuronal survival, reduced neuroinflammation, enhanced tissue repair, and improved behavioral recovery, highlighting the therapeutic potential of combining biomaterial engineering with optimized differentiation strategies [61].

Alongside these biophysical approaches, optimization of biochemical induction protocols has remained an active area of investigation. Classical differentiation media containing EGF, bFGF, BDNF, GDNF, NGF, retinoic acid, forskolin, IBMX, and other small molecules continue to represent the most widely employed strategies for neural induction [45,46,50,62].

A recent study by Silva-Lucero et al. (2026) developed a chemically defined and serum-free differentiation protocol to promote the acquisition of a neuronal-like phenotype in human dental pulp stem cells (DPSCs). DPSCs cultured in a medium supplemented with GDNF rapidly acquired neuron-like morphology characterized by elongated cellular processes and increased expression of mature neuronal markers, including NeuN and Neurofilament medium chain (NF-M). GDNF-treated cells showed some functional changes, such as hyperpolarized resting membrane potentials, typical of neural precursors or early neuronal maturation. Notably, the use of a chemically defined medium minimized the variability associated with serum-containing protocols and allowed the authors to demonstrate that GDNF alone was sufficient to drive neurogenic commitment [63].

However, considerable variability persists depending on MSC tissue source and species. Addressing this issue, Prpar Mihevc et al. (2020) systematically compared different neuronal differentiation protocols in canine MSCs derived from bone marrow and adipose tissue. The study demonstrated that protocol composition significantly influenced differentiation efficiency, with sequential exposure to growth factors producing more consistent neuronal morphology and increased expression of neuronal markers compared with simpler chemical induction methods. Nevertheless, the authors also emphasized that the acquisition of neuronal morphology does not necessarily correlate with complete functional neuronal maturation, underscoring the need for standardized differentiation protocols and rigorous functional validation [64].

In parallel with improvements in differentiation protocols, increasing attention has been devoted to post-transcriptional regulation through neurogenic microRNAs, particularly miR-124, which promotes neuronal lineage commitment by remodeling transcriptional networks. Likewise, extracellular vesicles derived from neuronal or glial cells have emerged as important mediators of paracrine neurogenic signaling, further expanding the molecular toolbox available for MSC programming [65]. The major technological advance has been achieved through the development of non-viral gene delivery systems capable of engineering MSCs with high efficiency while maintaining cell viability. In particular, a serum-tolerant biomimetic polymeric complex, APOs@BP composed of an apolipoprotein (APO) corona and a boronated polyethyleneimine (BP) core, for the genetic modification of human UC-MSCs was developed. Sequential intracellular delivery of an all-trans retinoic acid aminated derivative (atRAN) and miR-124 activated the PI3K–Akt, MAPK, and cGMP–PKG pathways, resulting in neuronal morphology, expression of mature neuronal markers, and acquisition of electrophysiological properties. Following transplantation into an APP/PS1 mouse model of Alzheimer’s disease, engineered MSCs exhibited enhanced neuronal integration, synapse formation, and significant improvements in cognitive performance, illustrating the translational promise of combining biomaterial-assisted gene delivery with developmental programming.

Beyond enhancing neuronal differentiation, recent studies have also investigated combinatorial strategies to improve the regenerative microenvironment after neural injury. Khodir et al. (2024) demonstrated that administration of human UC-MSCs together with melatonin significantly improved peripheral nerve regeneration in a rat sciatic nerve injury model compared with either treatment alone. Combined therapy reduced oxidative stress and inflammation, enhanced Schwann cell activity and axonal regeneration, increased expression of regeneration-associated proteins, and ultimately resulted in superior functional and histological recovery. Although this study did not specifically focus on neuronal differentiation, it highlights the importance of modulating the host microenvironment to maximize the regenerative potential of MSC-based therapies [66].

Collectively, these studies support a contemporary view of MSC neurogenesis as a multiscale process governed by the coordinated integration of extracellular matrix mechanics, membrane nanodomain organization, biomaterial-derived physical signals, developmental morphogens, epigenetic regulators, and advanced gene engineering technologies. Rather than acting independently, these regulatory mechanisms converge to control transcriptional programs, cytoskeletal remodeling, metabolic adaptation, and ultimately neuronal maturation. This integrated paradigm is progressively replacing conventional growth factor-based differentiation protocols and provides the conceptual framework for the next generation of MSC-based neuroregenerative therapies, in which biomaterials, mechanobiology, and precision genetic engineering are combined to generate functionally mature neurons with improved reproducibility and clinical translational potential.

Despite the increasing number of strategies developed to induce neuronal differentiation of MSCs, no single approach currently provides an optimal combination of differentiation efficiency, reproducibility, functional maturation, scalability, and translational safety. Conventional biochemical induction protocols based on growth factors, neurotrophic factors, retinoic acid, cyclic AMP modulators, and small molecules are relatively simple and experimentally accessible, and their composition can be adjusted to favor specific neuronal phenotypes. Main limitations include a substantial variability of outcomes, depending on MSC tissue source, donor, species, passage number, and culture conditions, as well as the frequent use of complex or poorly defined culture media. Moreover, increased expression of neuronal markers or acquisition of neuron-like morphology does not necessarily indicate the generation of functionally mature neurons, making electrophysiological and functional validation essential. Thus, although biochemical protocols are currently among the most readily implementable approaches, their lack of standardization represents a major obstacle to reproducibility and clinical translation.

Biophysical and biomaterial-based strategies offer several potential advantages, more closely reproducing the mechanical, structural, and physicochemical characteristics of the neural microenvironment. Control of matrix stiffness, membrane organization, electrical properties, and 3D extracellular matrix architecture can provide sustained and spatially organized cues that are difficult to achieve using soluble factors alone. These approaches may also reduce the dependence on high concentrations of exogenous differentiation factors and can enhance neuronal maturation and functional properties. Nevertheless, biomaterial-based systems introduce additional variables, including material composition, batch-to-batch variability, surface chemistry, mechanical properties, degradation kinetics, and 3D architecture, which can themselves affect cell behavior.

Genetic and post-transcriptional approaches, including microRNA delivery, non-viral gene engineering, and biomaterial-assisted intracellular delivery, provide a further level of control by directly manipulating the transcriptional and signaling networks underlying neuronal commitment. These strategies can potentially lead to more robust and sustained neurogenic programming than conventional soluble-factor protocols and may allow the generation of specific neuronal subtypes through targeted modulation of developmental pathways. However, their translation is constrained by challenges related to delivery efficiency, temporal control of transgene or RNA activity, off-target effects, cellular heterogeneity, and long-term safety. Although non-viral systems offer important advantages over integrating viral vectors, the reproducibility of nanoparticles or polymer-based delivery remains dependent on formulation and manufacturing parameters. In addition, combinatorial approaches that simultaneously manipulate intracellular signaling, microenvironmental properties, and gene expression may increase differentiation efficiency but also substantially increase process complexity and regulatory burden.

From a translational perspective, therefore, the principal challenge is not only to improve the percentage of MSCs expressing neuronal markers, but also to establish differentiation strategies that generate a homogeneous, stable, functionally mature, and clinically safe cell population. Biochemical approaches currently offer the greatest procedural simplicity, whereas biomaterial-based systems provide superior control over the cellular microenvironment and genetic or microRNA-based approaches offer greater molecular precision. However, these advantages are accompanied by increasing complexity and potential barriers to standardization. An important emerging direction is consequently the development of simplified combinatorial platforms in which a limited number of well-characterized biochemical signals are integrated with defined biophysical cues or transient molecular programming. Such approaches may provide a balance between differentiation efficiency and manufacturing feasibility while minimizing uncontrolled experimental variables. Future studies should therefore standardize MSC populations, culture conditions, neuronal subtype identity, long-term stability, electrophysiological functionality, and rigorous genomic and cellular safety. Establishing these parameters across independent laboratories and using clinically relevant manufacturing conditions will be essential for determining whether current MSC neurodifferentiation strategies can progress from proof-of-concept studies toward reproducible and therapeutically applicable platforms.

### 3.1. Subtype-Specific Neuronal Differentiation: Toward Dopaminergic- and Cholinergic-like Cells

Beyond generating generic neuron-like cells, recent differentiation protocols have increasingly focused on directing MSCs toward specific neuronal subtypes relevant to neurodegenerative disorders. Among these, dopaminergic neuron generation has attracted particular attention because of its potential application in Parkinson’s disease (PD), a disorder characterized by the progressive degeneration of dopaminergic neurons in the substantia nigra pars compacta.

Most protocols have been designed to recapitulate the developmental signaling pathways governing midbrain dopaminergic neurogenesis. The sequential activation of SHH and FGF8, which plays a central role during embryonic midbrain development, has therefore become the reference strategy for inducing dopaminergic commitment. This developmental paradigm promotes the expression of key transcription factors and phenotypic markers, including LMX1A/LMX1B, FOXA2, NURR1, PITX3, TH, dopamine transporter (DAT) and vesicular monoamine transporter-2 (VMAT2) [67]. Although these protocols have proven highly efficient in pluripotent stem cell-derived neural progenitors, their application to MSCs generally results in lower differentiation efficiency and incomplete functional maturation, reflecting the more restricted developmental potential of adult mesenchymal cells. Nevertheless, accumulating evidence indicates that the tissue source of MSCs profoundly influences their responsiveness to dopaminergic induction. Gaggi et al. (2020) demonstrated that human fetal membrane-derived MSCs (hFM-MSCs) retain the expression of pluripotency-associated genes, including OCT4, NANOG, SOX2, KLF4, OVOL1, and ESG1, suggesting a greater developmental plasticity than conventional adult MSC populations. Using a sequential differentiation protocol that reproduced embryonic midbrain development, the authors observed progressive induction of the dopaminergic transcription factors LMX1B, NURR1, and PITX3, followed by expression of mature dopaminergic markers including TH and DAT. The protocol consisted of a sequential induction using small-molecules on Matrigel, which involved the use of LDN193189 (BMP type I receptor inhibitor), SB431542 (TGF-β superfamily type I receptor inhibitor), SHH, purmorphamine, FGF8, and CHIR99021 (GSK-3 inhibitor). The culture medium was progressively transitioned to N2 supplement, followed by Neurobasal/B27 supplemented with neurotrophic and maturation factors. Differentiated cells acquired a morphology closely resembling primary mesencephalic dopaminergic neurons and maintained this phenotype for more than 40 days in culture. Although comprehensive electrophysiological characterization was not performed, these findings indicate that selected perinatal MSC populations possess an enhanced capacity for dopaminergic differentiation and may represent a promising cell source for PD-oriented regenerative strategies [68].

Several complementary strategies have also been explored to enhance dopaminergic differentiation efficiency. Han et al. (2021) demonstrated that a preinduction with a growth factor cocktail, followed by Caveolin-1 downregulation in human adipose-derived MSCs (AD-MSCs) significantly promoted neuronal differentiation after 29 days. This was indicated by the increased expression of dopaminergic markers, including TH and NURR1, suggesting that modulation of membrane-associated signaling pathways may facilitate dopaminergic lineage commitment [69]. Likewise, Absalan et al. (2021) adopted a multistep protocol. First, they generated neurospheres using DMEM/F12 supplemented with N2 for 2 days; in a second step, cells were plated in Matrigel or in monolayer cultures with a serum-free neurobasal medium added with human insulin-transferrin, sodium, bFGF and EGF for 6 days; in the last step, cells were incubated in N2/B27 with bFGF, FGF8b, SHH, and ascorbic acid for 6 days. Results were evaluated at day 2, 8, and 14. Compared with conventional two-dimensional (2D) cultures, it was found that AD-MSCs on Matrigel significantly enhanced dopaminergic differentiation, as indicated by the increased expression of neuronal and dopaminergic markers (TH, NURR1, LMX1A, and DAT). This supports the concept that extracellular matrix composition is a critical determinant of lineage specification [70]. Consistent with these observations, Moayeri et al. (2022) demonstrated that 3D culture conditions further improve dopaminergic differentiation of human UC-MSCs, after 20 days, by promoting neuronal morphology, neurite extension, and increased expression of dopaminergic markers, highlighting the importance of biomimetic microenvironments in recapitulating developmental cues [71].

More recently, chemically defined differentiation protocols have further improved dopaminergic conversion of MSCs. Liu et al. (2024) reported the direct chemical conversion of human UC-MSCs into dopaminergic neuron-like cells using the YFBP small-molecule cocktail, including Y27632, forskolin, SB431542, and SP600125 (ATP-competitive reversible JNK inhibitor). Differentiated cells expressed dopaminergic markers (TUBB3 and NeuN), exhibited neuron-like morphology, and displayed electrophysiological characteristics consistent with neuronal differentiation. Early conversion was reported after 7 days, with additional longer-term assessments, including day 28. Following transplantation into experimental Parkinsonian models, these cells contributed to behavioral improvement after 2 weeks; however, the authors emphasized that the observed therapeutic benefits likely resulted from the combined effects of neuronal differentiation together with the well-established trophic and immunomodulatory properties of MSCs, rather than unequivocal long-term neuronal replacement [72].

Collectively, these studies indicate that efficient generation of dopaminergic-like neurons from MSCs requires the integration of developmental morphogens, extracellular matrix-derived signals, 3D culture systems, and targeted modulation of intracellular signaling pathways. Although current protocols still produce cells that are generally less mature than pluripotent stem cell-derived dopaminergic neurons, they provide increasing evidence that subtype-specific neuronal programming of MSCs is feasible and may represent an important step toward disease-tailored regenerative therapies for Parkinson’s disease.

Beyond dopaminergic neurons, increasing efforts have been devoted to directing MSC differentiation toward other disease-relevant neuronal subtypes, although these approaches remain considerably less developed. Cholinergic neuron generation has attracted particular interest because of its potential application in Alzheimer’s disease and motor neuron disorders. Several differentiation protocols have combined developmental morphogens, including retinoic acid, SHH, NGF, EGF, and bFGF, to promote cholinergic specification. Using a three-step induction protocol based on retinoic acid and SHH, Sanooghi et al. (2021) demonstrated that human AD-MSCs acquired neuronal morphology together with increased expression of cholinergic markers, including acetylcholinesterase (AChE), following 14 days of differentiation [73]. Likewise, Stricker et al. (2021) generated neurospheres (at approximately 15–21 days) from human UC-MSCs cultured on a natural biopolymer membrane before inducing cholinergic differentiation with NGF, EGF, bFGF and B27 supplement for 11 days. The resulting cells expressed neuronal markers (βIII-tubulin and MAP2) together with the cholinergic enzyme ChAT, suggesting that biomaterial-assisted neural priming may improve cholinergic lineage commitment without prior genetic manipulation [74].

Among subtype-specific differentiation strategies, dopaminergic and cholinergic protocols are currently the most extensively characterized for MSCs, whereas reliable generation of GABAergic interneurons and spinal motor neurons remains largely confined to pluripotent stem cell systems and has yet to be reproducibly achieved in MSCs.

### 3.2. Toward Tissue-Specific Neural Differentiation: Lessons from Retinal Regeneration

The development of subtype-specific differentiation protocols has also been explored for retinal neural lineages, motivated by the limited regenerative capacity of the adult retina and the urgent need for effective therapies for retinal degenerative disorders such as glaucoma, retinitis pigmentosa, and age-related macular degeneration. Unlike dopaminergic differentiation, however, retinal lineage specification from MSCs remains at an earlier stage of development, with current protocols primarily generating retinal progenitor-like or retinal neuron-like phenotypes rather than fully mature retinal neurons.

Most retinal differentiation strategies have been designed to recapitulate key developmental events occurring during embryonic retinogenesis. Accordingly, combinations of developmental morphogens and growth factors, including Noggin, Dkk-1, bFGF, insulin-like growth factor-1 (IGF-1), retinoic acid, Activin A, and taurine, have been employed to modulate BMP, Wnt, FGF, and IGF signaling pathways, thereby promoting the expression of early retinal transcription factors such as PAX6, RAX (RX), VSX2 (CHX10), and OTX2, followed by markers associated with more differentiated retinal cell populations [75,76].

Among retinal cell types, retinal ganglion cell (RGC)-like differentiation has received the greatest attention because RGC degeneration represents the primary pathological event in glaucoma and other optic neuropathies. Under retinal induction conditions, MSCs acquire neuron-like morphology and express RGC-associated markers including BRN3A (POU4F1), Thy-1, MAP2, and neurofilament proteins. Similarly, protocols incorporating retinoic acid and taurine have promoted the expression of photoreceptor-associated proteins, including Cone-Rod Homeobox (CRX), recoverin, and rhodopsin, whereas alternative induction strategies have generated retinal pigment epithelium-like cells expressing RPE65 and bestrophin [75].

In addition to classical growth factor-based approaches, more recent studies have explored bioengineering and molecular programming strategies to promote retinal neurogenic commitment. Three-dimensional scaffold-based systems have been proposed to better mimic the retinal microenvironment, as illustrated by fibrin-based encapsulation of conjunctiva-derived MSCs, which was suggested as a potential platform for retinal tissue engineering and structural organization. Photoreceptor-like cell differentiation was induced by exposing cells to taurine for 14 days. The expression of photoreceptor-associated markers, including rhodopsin, protein kinase C (PKC), CRX, peripherin, recoverin, nestin, and Retinal Pigment Epithelium-Specific 65 kDa Protein (RPE65), was assessed. Compared with the 2D group, the 3D fibrin hydrogel group showed higher cell viability and enhanced photoreceptor-like differentiation [77,78].

Genetic engineering strategies have been further explored to enhance retinal differentiation efficiency, as shown by EPO-modified human Wharton’s jelly-derived MSCs (WJ-MSCs). When cultured for 2 weeks in an induction medium containing taurine and retinoic acid, these cells exhibited increased expression of photoreceptor-associated markers, particularly Con-Rod Homeobox (CRX) and Rhodopsin (RHO), under in vitro differentiation conditions. It is suggested that EPO engineering may enhance commitment toward a rod photoreceptor-like phenotype, although without evidence of full functional maturation [79].

Similarly, post-transcriptional regulation via microRNAs has emerged as a critical mechanism in retinal fate specification. Overexpression of the miR-183/96/182 cluster in human BM-MSCs induced a retina-like phenotype characterized by upregulation of retinal lineage-associated genes after 24 and 48 h from transfection, reflecting partial activation of retinal developmental programs [80]. In a complementary approach, miR-9-1 overexpression in conjunctiva-derived MSCs cultured on a 3D nanofibrous scaffold promoted photoreceptor-like differentiation at days 14 and 21, further supporting the role of microRNA-driven regulatory networks in retinal neurogenic programming [81].

Collectively, these findings indicate that retinal neurogenic differentiation of MSCs can be enhanced through the integration of developmental morphogens, biomaterial-based microenvironments, genetic engineering, and microRNA-mediated regulation. However, despite these advances, the resulting cells predominantly exhibit partial retinal-like phenotypes, and there is still no robust evidence of stable functional maturation, synaptic integration, or faithful recapitulation of retinal neuronal circuitry. Therefore, retinal differentiation of MSCs should currently be interpreted as a process of incomplete retinal lineage priming rather than full neurogenic conversion.

Importantly, the field remains incomplete and still evolving, and further studies are required to better define the molecular mechanisms governing retinal lineage commitment, to improve the efficiency and reproducibility of differentiation protocols, and to establish standardized functional assays capable of assessing true neuronal maturation (Table 1).

## 4. Extracellular Vesicles and Cell-Free Therapies

MSCs secrete a broad spectrum of trophic molecules and extracellular vesicles (EVs) containing neurogenic proteins, messenger RNAs, and microRNAs capable of modulating neuronal differentiation and promoting neural tissue repair through autocrine and paracrine mechanisms [82,83]. Recent studies have further highlighted the relevance of both conditioned media and MSC-derived EVs as cell-free therapeutic approaches in neurological disorders. Using conditioned media obtained from human AD-MSCs, Baldassarro et al. (2023) demonstrated significant neuroprotective and promyelinating activity in in vitro models of neural damage. Importantly, the authors observed marked inter-donor variability in the biological efficacy of the conditioned media, indicating that the therapeutic potential of the MSC secretome may vary considerably among individual cell donors. While all conditioned media samples preserved the mesenchymal phenotype of the parental cells, only selected preparations effectively promoted oligodendrocyte differentiation, myelin protein expression, and neuronal survival, emphasizing the need for standardized potency assays when developing secretome-based therapies [84]. The importance of controlling secretome composition was further highlighted by He et al. (2023), who developed an acoustofluidic platform to investigate how mechanical stimulation influences MSC secretory activity. The study demonstrated that exposure of MSCs to acoustically generated mechanical cues significantly altered the composition and biological properties of the secretome, enhancing the release of regenerative and immunomodulatory factors. These findings provided direct evidence that mechanobiological signals can be exploited to engineer MSC secretory profiles, suggesting that physical conditioning strategies may represent a powerful approach to generate more reproducible and therapeutically effective secretome products [85]. More recently, Yang et al. (2024) investigated the effects of MSC-derived extracellular vesicles in a mouse model of intracerebral hemorrhage. The authors demonstrated that EV administration reduced neuronal injury and improved neurological function by suppressing ferroptosis, a form of iron-dependent regulated cell death increasingly implicated in neurodegeneration. Mechanistically, EV treatment was associated with reduced lipid peroxidation, decreased iron accumulation, and modulation of ferroptosis-related proteins within the injured brain tissue, supporting a direct role of MSC-derived vesicles in regulating neuronal survival pathways following hemorrhagic injury [86].

Interestingly, even in the field of ocular diseases, the exploitation of the paracrine regenerative properties of MSCs has gained increasing interest over the last ten years. In a landmark study Deng et al. (2021) demonstrated that MSC-derived exosomes protected photoreceptors from degeneration by transferring miR-21, thereby suppressing apoptosis and preserving retinal structure and function in experimental retinal degeneration. Rather than supporting direct neuronal replacement, these findings identified extracellular vesicle-mediated signaling as a major mechanism underlying MSC-mediated retinal neuroprotection [87]. Consistent with this concept, Lin et al. have further shown that MSC transplantation modulates retinal inflammation, attenuates microglial activation, promotes retinal ganglion cell survival, and preserves visual function through trophic support and immune regulation [88]. Further evidence for the neuroprotective potential of MSC-derived EVs was provided by Yu et al. (2024), who engineered MSCs to overexpress miR-22-3p and subsequently isolated small extracellular vesicles enriched in this microRNA. In experimental models of retinal ganglion cell injury, these vesicles significantly enhanced neuronal survival and attenuated apoptosis. Mechanistic analyses revealed that the protective effects were mediated through regulation of the MAPK signaling pathway, identifying miR-22-3p as a functional cargo capable of modulating intracellular neuroprotective responses. Notably, EVs derived from miR-22-3p-overexpressing MSCs exhibited stronger neuroprotective effects than vesicles obtained from unmodified MSCs [89].

Even though accumulating evidence demonstrates that the secretome of naïve MSCs possesses remarkable neuroprotective, immunomodulatory, and regenerative properties, its therapeutic potential may represent only part of the broader biological capabilities of these cells. Indeed, most studies conducted to date have focused on secreted factors and extracellular vesicles derived from undifferentiated MSC populations. In contrast, relatively little is known about the composition and biological activity of the secretome released by MSCs following neuronal differentiation. Given the profound transcriptional, epigenetic, and metabolic changes that accompany neurogenic commitment, it is conceivable that neuronally differentiated MSCs may produce a distinct repertoire of trophic factors, extracellular vesicles, and regulatory RNAs more closely tailored to the requirements of neural tissue repair. Such a specialized neurogenic secretome could potentially provide enhanced support for neuronal survival, neurite outgrowth, synaptic plasticity, remyelination, and circuit reconstruction compared with naïve MSC-derived products. We believe that future investigations could help bridge the existing knowledge gap regarding the therapeutic potential of EVs derived from neuronally differentiated MSCs.

## 5. Clinical Translation

Clinical translation of MSC-based therapies has expanded considerably over the past decade, with clinical trials evaluating their therapeutic potential in a broad range of neurological disorders, including multiple sclerosis, Parkinson’s disease, Alzheimer’s disease, ischemic stroke, traumatic brain injury, spinal cord injury, amyotrophic lateral sclerosis, multiple system atrophy and cerebral palsy [22,90]. Importantly, the vast majority of these studies (Table 2) have employed naïve MSCs derived from bone marrow, adipose tissue or umbilical cord rather than MSC-derived neuron-like cells. In particular, a multicentre, randomized, double-blind, placebo-controlled phase I/II cross-over study was designed to evaluate the safety and exploratory efficacy of intravenously administered autologous BM-MSCs in patients with active multiple sclerosis. The study employed naïve, non-differentiated MSCs. Importantly, the trial was designed to standardize MSC manufacturing, dosing, and imaging analysis across multiple international centers rather than to assess neuronal differentiation or regenerative integration within the central nervous system. However, the publication reported the study protocol rather than clinical outcomes [91].

In ALS patients autologous BM-MSCs induced to secrete neurotrophic factors (MSC-NTF) demonstrated a favorable safety profile and biological activity, although definitive clinical efficacy has not yet been established [92,93]. Similarly, in chronic stroke, intracerebral transplantation of transiently modified BM-MSCs by transfection with a plasmid containing the human Notch-1 intracellular domain, was associated with long-term safety and improvements in neurological function [94]. In Alzheimer’s disease, intraventricular administration of UC-MSCs was shown to be feasible and generally well tolerated, with only transient inflammatory responses observed following transplantation [95]. More recently, a phase 1 clinical trial of autologous AD-MSCs in the treatment of paralysis due to traumatic spinal cord injury reported that intrathecal administration of autologous AD-MSCs was safe and associated with neurological improvements in a subset of treated individuals [96]. Importantly, while neuronal differentiation of MSCs remains an active area of experimental investigation, its translation into clinical practice has yet to be achieved. In fact, all published clinical studies conducted to date have employed naïve MSCs or MSCs engineered to enhance neurotrophic factor secretion. No clinical trial has yet evaluated MSC-derived dopaminergic, cholinergic, GABAergic or other neuronally differentiated cell populations as a cell replacement strategy for neurological disorders. This highlights a substantial translational gap between advances in MSC neurogenic differentiation achieved in preclinical models and their current clinical implementation.

## 6. Challenges and Future Perspectives

Despite substantial advances in the neuronal differentiation of MSCs, several biological, technical, and translational challenges continue to limit the development of clinically effective MSC-derived neuronal therapies. Although numerous protocols have successfully generated neuron-like cells expressing neuronal markers and exhibiting morphological features consistent with neural differentiation [63,97], the production of fully mature, functionally integrated, and therapeutically effective neuronal populations remains a fundamental goal yet to be achieved. Addressing these limitations will be essential for the successful translation of MSC-derived neuronal cells from experimental studies to clinical applications. Among the differentiation protocols reported, there is currently no consensus on the optimal protocol for generating specific neuronal subtypes. Future efforts should focus on establishing standardized differentiation procedures and internationally accepted quality control criteria to improve reproducibility and facilitate regulatory approval.

Another important challenge concerns the substantial heterogeneity observed within differentiated MSC populations. Even under identical culture conditions, only a subset of cells often acquires neuronal characteristics, resulting in mixed populations composed of partially differentiated cells, residual MSCs, and cells exhibiting intermediate phenotypes. Such heterogeneity may compromise therapeutic efficacy and complicate quality control procedures required for clinical applications [98,99].

Successful clinical translation requires not only the generation of neuronal-like cells in vitro but also their survival, integration, and functionality following transplantation. The hostile microenvironment present in neurodegenerative diseases and traumatic injuries may expose transplanted cells to inflammation, oxidative stress, excitotoxicity, and hypoxia, leading to substantial post-transplantation cell loss [100]. Even when transplanted cells survive, integration into existing neural circuits remains a formidable challenge. Functional recovery requires the establishment of appropriate synaptic connections, axonal growth toward target tissues, and long-term participation in neural network activity. To date, convincing evidence demonstrating extensive functional integration of MSC-derived neuronal cells in vivo remains limited. In addition, the long-term stability of neuronal differentiation remains poorly understood. The development of strategies capable of stabilizing neuronal identity will therefore be essential for future clinical applications.

Finally, growing evidence suggests that many therapeutic effects attributed to MSCs are mediated by extracellular vesicles and exosomes. These cell-free approaches may elude several limitations associated with cell transplantation, including poor survival, immune rejection, and tumorigenic concerns. Furthermore, engineered exosomes carrying neurogenic factors, microRNAs, or therapeutic molecules may represent a novel generation of neuroregenerative therapies.

## 7. Conclusions

The field of MSC neuronal differentiation has evolved considerably over the last decade, transitioning from relatively simple chemical induction protocols to sophisticated approaches integrating developmental biology, biomaterials and genetic engineering. Nevertheless, significant challenges remain before MSC-derived neuronal products can become clinically viable therapies.

Future success will likely depend on the convergence of multiple disciplines, including stem cell biology, neurodevelopment and bioengineering. Standardized differentiation protocols, rigorous functional validation, advanced manufacturing strategies, and carefully designed clinical trials will be essential to determine whether MSC-derived neuronal cells can ultimately fulfill their promise as regenerative therapies for neurological disorders.

## Figures and Tables

**Table 1:** Overview of neural differentiation approaches of mesenchymal stem/stromal cells (MSCs). Different neural fates obtained from MSCs of distinct tissue sources are summarized, together with the differentiation strategies and protocols adopted and the markers used to characterize the neural phenotype. **Abbreviations**: AChE, Acetylcholinesterase; ChAT, Choline Acetyltransferase; CRX, Cone-Rod Homeobox; DAT, Dopamine Transporter; EGF, Epidermal Growth Factor; EPO, erythropoietin; FGF8, Fibroblast Growth Factor 8; hAD-MSCs, Human adipose-derived MSCs; hBM-MSCs, Human bone marrow-derived MSCs; hCJ-MSC, Human Conjunctiva-derived MSCs; bFGF, Basic Fibroblast Growth Factor; hFM-MSCs, Human fetal membrane-derived MSCs; hUC-MSCs, Human umbilical cord-derived MSCs; hWJ-MSCs, Human Wharton’s jelly-derived MSCs; LMX1A, LIM Homeobox Transcription Factor 1 alfa; LMX1B, LIM Homeobox Transcription Factor 1 Beta; MAP2, Microtubule-Associated Protein 2; NeuN, Neuronal nuclei; NGF, Nerve Growth Factor; NURR1, Nuclear Receptor Subfamily 4 Group A Member 2; PITX3, Paired-Like Homeodomain Transcription Factor 3; PKC, Protein Kinase C; RA, retinoic acid; RPE65, Retinal Pigment Epithelium-Specific 65 kDa Protein; SHH, Sonic Hedgehog; TUBB3, βIII-tubulin; TH, Tyrosine Hydroxylase.

Differentiation Type	MSC Source	Differentiation Strategy	Functional Validation	Reference
Dopaminergic neuron-like cells	hFM-MSCs	Sequential small-molecule induction on Matrigel designed to reproduce embryonic midbrain development, using LDN193189, SB431542, SHH, purmorphamine, FGF8, and CHIR99021. The protocol involved progressive induction of LMX1B, NURR1, and PITX3, followed by the expression of mature dopaminergic markers TH and DAT. The phenotype for more than 40 days.	Electrophysiological characterization was not comprehensively performed.	[68]
Dopaminergic neuron-like cells	hAD-MSCs (passage 5)	Preinduction with a growth factor cocktail, followed by Caveolin-1 downregulation. The expression of the dopaminergic markers TH and NURR1, evaluated after 29 days of differentiation, was enhanced.	Not reported	[69]
Dopaminergic neuron-like cells	hAD-MSCs	By a multistep protocol, the study compared differentiation outcomes between Matrigel-based and conventional 2D culture conditions. In Matrigel cultures, increased expression of neuronal and dopaminergic markers, including TH, NURR1, LMX1A, and DAT, was observed.	Not reported	[70]
Dopaminergic neuron-like cells	hUC-MSCs	Three-dimensional (3D) cultures in Matrigel-coated plates, incubated with a cocktail of dopaminergic differentiation factors for 20 days. Neuronal morphology was improved, along with neurite extension, and expression of dopaminergic markers.	Not reported	[71]
Dopaminergic neuron-like cells	hUC-MSCs passages 3 to 6	Direct chemical conversion using the YFBP small-molecule cocktail. Differentiated cells expressed dopaminergic markers (TUBB3 and NeuN), exhibited neuron-like morphology, after 7 and 28 days. Following transplantation into experimental Parkinsonian models, the cells contributed to behavioral improvement after 2 weeks.	Electrophysiological characteristics compatible with neuronal differentiation Presence of typical action potentials and sodium and potassium ion channel activity.	[72]
Cholinergic neuron-like cells	hAD-MSCs	Three-step induction protocol based on RA and SHH, resulting in neuronal morphology and increased expression of the cholinergic marker AChE after 14 days post-induction.	Not reported	[73]
Cholinergic neuron-like cells	hUC-MSCs	Generation of neurospheres (at approximately 15–21 days) from MSCs cultured on a natural biopolymer membrane, followed by cholinergic induction with NGF, EGF, bFGF and B27 for 11 days. Resulting cells expressed βIII-tubulin, MAP2, and ChAT.	Not reported	[74]
Photoreceptor progenitor-like cells	hCJ-MSC passages 3	Photoreceptor-like cell differentiation was induced by exposing cells to taurine for 14 days in either conventional tissue culture plates (2D) or a 3D fibrin hydrogel scaffold. The expression of photoreceptor-associated markers, including rhodopsin, PKC, CRX, peripherin, recoverin, nestin, and RPE65, was assessed. The 3D fibrin hydrogel group showed higher cell viability and enhanced photoreceptor-like differentiation compared with the 2D group.	Not reported	[78]
Photoreceptor-like cells	hWJ-MSCs	Genetic modification with EPO, which increased the expression of photoreceptor-associated markers after induction for 2 weeks with medium containing taurine and retinoic acid.	No evidence of full functional maturation	[79]
Retina-like phenotype	hBM-MSCs Passages 3 to 4	Overexpression of the miR-183/96/182 cluster, inducing upregulation of retinal lineage-associated genes and a retina-like phenotype. Gene expression analyses were performed 24 and 48 h after transfection.	Not reported	[80]
Photoreceptor-like cells	hCJ-MSC	miR-9-1 overexpression in MSCs cultured on a 3D nanofibrous scaffold, promoting photoreceptor-like differentiation. The evaluations were carried out at days 14 and 21.	Not reported	[81]

**Table 2:** Clinical studies on mesenchymal stromal cell (MSC)-based therapies in neurological disorders. **Abbreviations:** AD, Alzheimer’s disease; AD-MSCs, adipose-derived mesenchymal stromal cells; ALS, amyotrophic lateral sclerosis; BM-MSCs, bone marrow-derived mesenchymal stromal cells; CSF, cerebrospinal fluid; MSC-NTF, neurotrophic factor-secreting mesenchymal stromal cells; UC-MSCs, umbilical cord-derived mesenchymal stromal cells.

Therapeutic Approach	Disease Indication	MSC Source	Administration	Clinical Phase	Study Objectives	Outcomes	Ref.
Naïve MSCs	Multiple sclerosis	Autologous BM-MSCs	Intravenous administration (single dose)	Phase I/II multicentre, randomized, double-blind, placebo-controlled crossover trial (MESEMS).	To evaluate safety and efficacy on brain MRI within the first 24 weeks after treatment.	Clinical outcomes are not reported in the protocol publication.	[91]
MSCs engineered to enhance neurotrophic-factor secretion (MSC-NTF)	ALS	Autologous BM-MSCs	Intrathecal administration (single dose)	Phase II double-blind, placebo-controlled trial	The study was designed to evaluate safety, clinical outcomes, and biomarkers.	MSC-NTF cells were safe and well tolerated. Increased CSF neurotrophic factor levels and decreased CSF inflammatory biomarker levels were observed following transplantation. Early signs of efficacy were reported in a prespecified subgroup of rapid progressors	[92]
MSCs engineered to enhance neurotrophic-factor secretion (MSC-NTF)	ALS	Autologous BM-MSCs	Intrathecal administration (3 doses)	Phase III randomized, placebo-controlled trial	To evaluate clinical efficacy.	MSC-NTF cells were safe and well tolerated. Significant improvements in CSF biomarkers. Although not significant, preliminary signals of clinical benefit were observed.	[93]
Genetically modified MSCs (transfection with a plasmid encoding the human Notch-1 intracellular domain)	Chronic stroke	BM-MSCs	Intracerebral implantation	Phase I/IIa clinical study	To evaluate safety and tolerability, alongside exploratory neurological and functional outcomes.	Favorable safety findings were reported and improvements in neurological function during long-term follow-up.	[94]
Naïve MSCs	AD	UC-MSCs	Intraventricular administration (3 doses)	Phase I clinical study	To evaluate the safety and dose-limiting toxicity	Generally well tolerated; transient inflammatory responses were reported following transplantation.	[95]
Naïve MSCs	Traumatic spinal cord injury	Autologous AD-MSCs	Intrathecal administration (single dose)	Phase I clinical study	To evaluate the safety of intrathecal administration.	No serious adverse events were reported. Neurological improvements were reported in a subset of participants.	[96]

## Data Availability

No new data were created or analyzed in this study. Data sharing is not applicable to this article.
